# Investigation of the Influence of PLA Molecular Structure on the Crystalline Forms (α’ and α) and Mechanical Properties of Wet Spinning Fibres

**DOI:** 10.3390/polym9010018

**Published:** 2017-01-06

**Authors:** Michał Puchalski, Sylwia Kwolek, Grzegorz Szparaga, Michał Chrzanowski, Izabella Krucińska

**Affiliations:** Department of Material and Commodity Sciences and Textile Metrology, Faculty of Material Technologies and Textile Design, Centre of Advanced Technologies of Human-Friendly Textiles “Pro Humano Tex”, Lodz University of Technology, Zeromskiego 116, 90-924 Lodz, Poland; kwolek.sylwia@outlook.com (S.K.); grzegorz.szparaga@p.lodz.pl (G.S.); michal.chrzanowski@p.lodz.pl (M.C.); izabella.krucinska@p.lodz.pl (I.K.)

**Keywords:** polylactide, crystallisation, crystalline forms, wet spinning, fibres, WAXD, DSC

## Abstract

In this paper, the influence of the molecular structure of polylactide (PLA)—characterised by its molar mass and content of d-lactide isomer—on the molecular ordering and α’–α form transition during fibre manufacturing by the wet spinning method is described. Fibres were studied by wide-angle X-ray diffraction (WAXD) and differential scanning calorimetry (DSC). Additionally, the physical and mechanical properties of the fibres were determined. This study also examines the preliminary molecular ordering and crystallisation of PLA fibres at various draw ratios. The performed experiments clearly show the dependence of the molecular ordering of PLA on the molar mass and d-lactide content during the wet spinning process. The fibres manufactured from PLA with the lowest content of d-lactide and the lowest molar mass were characterised by a higher tendency for crystallisation and a higher possibility to undergo the disorder-to-order phase transition (α’ to α form). The structural changes in PLA explain the observed changes in the physical and mechanical properties of the obtained fibres.

## 1. Introduction

In recent years, poly(lactic acid), also called polylactide (PLA), has become the most commonly used biodegradable material produced from completely renewable sources such as corn, sugar, and vegetables. Among the advantages of this polymer, its excellent mechanical properties—which are comparable to those of other conventional polymers produced from petroleum sources—allow for their facile adaptation to current well-known processing technologies used today [1]. The synthesis of PLA is based on the polycondensation of lactic acid (poly(lactic acid)) or the ring-opening polymerisation of lactide obtained from the depolymerisation of oligomers of lactic acid (polylactides), which is a product of the fermentation of biomass such as corn [2,3]. However, due to high manufacturing costs, the use of PLA was limited to medical applications for many years [4,5]. A decrease in the price of PLA has expanded the range of possible applications. For example, PLA has become a major starting material in the manufacture of biodegradable textiles [6,7]. The preparation, structure, and properties of products made of PLA and its derivatives are the subjects of intensive scientific and technological investigations [8,9,10,11]. The physical properties, in combination with biodegradability, makes polylactide fibres and nonwovens fabrics particularly useful raw materials for the preparation of disposable medical and hygiene textiles [12,13,14]. Other applications include technical textiles used in filtration or agriculture [15,16].

The physical and chemical properties of the final products obtained from PLA depend on the various stereoisomeric lactide forms and the formation of different supramolecular structures of the polymers. The properties of PLA also depend on the presence of units with different chirality in the polymer chain. Mixed chirality in PLA reduces its ability to crystallise, which, in the case of PLA, strongly influences the useful properties of the final product and its degradation. The poly(l-lactide) homopolymer is a crystallisable polymer that can crystallise in three different polymorphs, α, β, and γ, which can be obtained under specific crystallisation conditions. The most frequently observed forms are the orthorhombic (parameters of cell: *a* = 1.05 nm, *b* = 0.610 nm, *c* = 2.880 nm, and α = β = γ = 90°) or pseudo-orthorhombic (*a* = 1.060 nm, *b* = 0.610 nm, *c* = 2.880 nm, and α = β = γ = 90°) α form, which crystallise from melt or solution under normal conditions [17]. The β and γ forms are less often observed in poly(l-lactide). The trigonal (*a* = 1.052 nm, *b* = 1.052 nm, *c* = 0.880 nm, and α = β = 90°, and γ = 120°) or orthorhombic (*a* = 1.031 nm, *b* = 1.821 nm, *c* = 0.900 nm, and α = β = γ = 90°) β form of PLA is produced by stretching the α form at very high drawing ratio and high temperature [18]. The orthorhombic γ form (*a* = 0.995 nm, *b* = 0.625 nm, *c* = 0.880 nm, and α = β = γ = 90°) is produced through epitaxial crystallisation [19]. 

Pan et al. in 2007 [20] and Zhang et al. in 2008 [21] presented that, in poly(l-lactide) homopolymer, the disordered α’ and the ordered α polymorphs are formed during crystallisation below 100 °C and above 120 °C, respectively. However, in the intermediate temperature range from 100 to 120 °C, both forms crystallise. The content of α form increases, whereas that of the α’ form decreases, with increasing crystallisation temperature. Moreover, the disorder-to-order phase transition (α’ to α form) is observed during heating. It is worth noting that the difference between the α’ and α cell parameters is minor; the cell parameters c and b of α’ are similar to those of α. Only the cell parameter a of α’ (1.072 nm) is larger than that of α. The influence of the structural difference between α and α’ crystals on the macroscopic parameters of textile materials, such as tensile strength, has not been clearly reported.

Enough information is clearly not available in the literature regarding the conditions required to induce disorder-to-order phase transitions, with associated changes in the physical and mechanical properties [22,23]. Most of the data in the literature show the impact of technological parameters; for example, the impact of those used in the process of spinning from the melt on the crystallisation of the polymer and the resulting mechanical properties. The complementary investigation of the influence of technological parameters on the crystallisation, disorder-to-order phase transition, and its effect on the properties of PLA products, for example, was reported on the spun-bonded nonwoven forming process [24]. The complete analysis of the influence of the calendering temperature on tensile strength with the analysis of supramolecular changes allowed a scientific explanation of the phenomenon observed in the technological process and allowed optimal parameters of such a process to be found. In this paper, the results of the investigation of the influence of the molecular structure of commercially available polylactide on the technological process of wet spinning fibres and the crystallisation of PLA during that process are presented. The studied polymers were characterised by molar mass and d-lactide isomer content, which are the molecular properties responsible for the physical properties—including the crystalline structure—of the final product. The obtained experimental results also clarify which molecular structures are suitable to produce fibres by wet spinning and evaluate whether this method of spinning fibres includes technological parameters that allow for the observation of the disorder-to-order phase transition and its influence on the mechanical properties of the fibres. The analysis was performed using analytical techniques such as wide-angle X-ray diffraction (WAXD), differential scanning calorimetry (DSC), and tests of the physical properties of the fibres according to standard methods.

## 2. Materials and Methods

### 2.1. Raw Material

Fibres were obtained from commercially available poly(l-lactide) from Nature Works LLC (Minnetonka, MN, USA) with various d-lactide contents and molar masses. The content of the d-lactide isomer was taken from the Nature Works Data, and the molar mass was determined by size-exclusion chromatography (SEC, Wyatt Technology Corporation, Santa Barbara, CA, USA) with a multi-angle light scattering (MALLS) detector in methylene chloride (Table 1).

### 2.2. Rheological Properties of Spinning Solutions

The rheological properties of PLA solutions in dichloromethane (POCH, Gliwice, Poland) were determined using a RheolabQC (Anton Paar GmbH, Graz, Austria) rotary rheometer at 20 °C with an H-type cylinder. The rheological parameters “*n*” and “*k*” were estimated on the basis of the flow curves according to the Ostwald–de Waele equation:

(1) $$\tau =k\cdot \gamma^{n}$$

where τ is a shear stress, and γ is shear rate.

### 2.3. Wet Spinning Method

**Figure 1:**
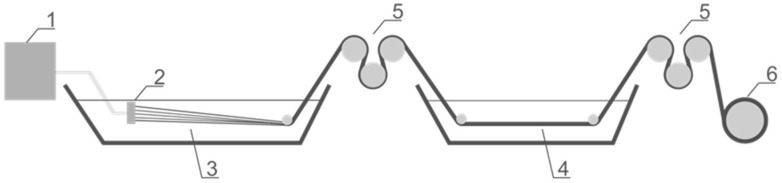
The scheme of the used wet spinning system contained: (1) feeding tank and pump; (2) spinneret; (3) coagulation bath; (4) drawing bath; (5) winder system; (6) take up spool.

The PLA fibres were prepared using a laboratory technological stand. The multifilament fibres were spun using a spinneret with 250 holes with 80 μm diameters and a spinning velocity of 1 m/min. The coagulation process was carried out in a bath containing a solution of ethanol and dichloromethane (90:10) at 10 °C. The drawing process was performed in a water bath at 45 °C using a godet system (Figure 1). Each polymer spinning process was carried out to obtain the maximum draw ratio.

### 2.4. WAXD Structural Analysis

The supramolecular structures of the PLA samples were determined by wide-angle X-ray diffraction (WAXD) using an X’Pert PRO diffractometer (CuKα source, λ = 0.154 nm) from PANalytical (Eindhoven, The Netherlands). The diffractograms for the powdered samples were recorded over a 2θ range of 5°–60° with a step 0.05°. Numerical analysis of the obtained WAXD data was performed using the WAXSFIT software.

### 2.5. Thermal Properties Analysis

Characterisation of the thermal properties of the PLA fibres was carried out by differential scanning calorimetry (DSC) using a DSC measurements Q2000 (TA Instruments, New Castle, DE, USA) instrument. The specimens were first heated from 0 to 250 °C, then cooled to −30 °C and immediately reheated to 250 °C at a rate of 10 °C/min. The measurements were collected under a nitrogen atmosphere. 

### 2.6. Methods of Physical Properties Measurements

The mechanical parameters of the studied fibres, such as stress at break and strain at break, were determined using an Instron 5511 (Instron, Norwood, MA, USA) mechanical testing machine. Measurements were carried out according to the PN EN ISO 2062:2010 standard. The linear density of the multifilament was measured by the use of a WTB 200 (Radwag, Radom, Poland) digital balance and the PN ISO 1973:1997 standard.

## 3. Results and Discussion

### 3.1. Influence of the PLA Molecular Structure on the Spinning Process

The wet spinning technique for stable solution processing into fibres requires an appropriate apparent dynamic viscosity of the polymer solution that depends on the concentration of the polymer solution. A low concentration of the spinning solution may adversely affect the continuity of the flow of the spinning solution, while an excess concentration can increase the apparent dynamic viscosity, which may disrupt the moulding process. The rheological parameters of the spinning solutions have a significant effect on both the flow rate distribution during the flow of the spinning solution in the spinneret channels and the longitudinal flow rate gradient along the fibre formation track. The value of the longitudinal flow rate gradient considerably affects the orientation of the structural fibre elements. The rheological parameters and the ideal concentration of the polymer solution strongly depend on the molecular structure of the polymer—in this case, the polylactide structure. 

**Figure 2:**
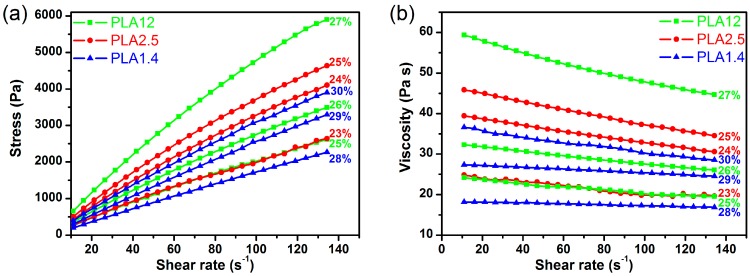
The rheological characteristics of selected polylactide solutions with different concentrations: (**a**) the relation between the apparent dynamic viscosity and the shear rate and (**b**) the flow curves.

In Figure 2, the rheological results of the selected polylactide solutions are presented. As can be observed, the studied solutions of PLA12 are shear-thinning non-Newtonian fluids without a flow limit, and the shear stress increases less than proportionally with the shear rate (Figure 2a). PLA2.5 is characterized by similar molar mass but lower d-lactide content, and similar rheological characteristics were recorded. The final studied polymer solutions prepared from PLA1.4 were characterised by similar rheological properties to the Newtonian fluid, and the shear stress increases nearly proportionally with the shear rate (Figure 2a). The apparent dynamic viscosity decreases with increasing shear rate, which is typical of polymer solutions (Figure 2b). Similar to the analysed shear stress characteristics, the increase in the apparent dynamic viscosity as a function of shear rate is less apparent for PLA1.4. 

According to the Ostwald–de Waele equation, the estimated rheological parameters “*n*” and “*k*” of the studied polylactide solutions are presented in Table 2. According to the presented results, the rheological parameter “*k*”, which is a factor of solution consistency, increases with increasing polymer concentration in the solution, which is typical of solutions of high molar mass compounds. However, the polymer concentration and the rheological parameter “*k*” clearly depend on the molar mass of polylactide. For PLA1.4, which is characterised by the lowest values of *M*_w_ and *M*_w_/*M*_n_, an apparent dynamic viscosity in the range of 20–40 Pa·s was obtained for concentrations above 28%, and for the PLA2.5 for concentration of approximately 23%. Notably, for commercially available polylactide, the content of d-lactide also influences the rheological parameter “*k*”. For PLA12, which has a similar molar mass to PLA2.5, higher concentrations (above 25%) are needed to obtain solutions with apparent dynamic viscosities in the range of 20–40 Pa·s.

The estimated values of the rheological parameter “*n*” show that the most non-Newtonian behaviour is observed for the solutions of PLA with 15% d-lactide isomer content, where “*n*” is approximately 0.7, while the least non-Newtonian behaviour is observed for the solution of PLA with the lowest molar mass. Additionally, analysis of the dependence of the rheological parameter “*n*” on the concentration of the solution revealed that the “*n*” parameter decreases with increasing polymer solution concentration, which is typical of solutions of high molar mass compounds.

The rheological analysis of polylactide solutions allowed for the determination of the spinning solution concentrations for which the apparent dynamic viscosity fell within the appropriate range for wet spinning from solution. These concentrations were 26%, 24%, and 29% for PLA12, PLA2.5, and PLA1.4, respectively. The wet spinning process was carried out using the same parameters for each of the spinning solutions (as-spun draw ratio of −10% and a head with 250 holes of 0.08 mm diameter) and varying only the total draw ratio. The variety of obtained fibres and their formation conditions are presented in Table 3.

The performed wet spinning process showed an influence of the molecular structure of the raw material on the forming and drawing of the fibres. The low molar mass and low content of d-lactide isomer allow for the preparation of fibre from PLA1.4 only using high total draw ratios in the range of 500%–700%. When the total draw ratio is below 500%, the tension of the fibre bundle was insufficient for carrying out a stable wet spinning process. Most likely, the necessary application of a high draw ratio is due to the rheological properties of the polymer solution being similar to those of a Newtonian liquid, which results from the polymer structure. For PLA2.5, which has a higher molar mass than PLA1.4 but similar d-lactide isomer content, the preparation of fibre using total draw ratios lower than 500% was possible. In contrast, the structural properties of the polymer, and thus the rheological properties of the spinning solution, did not allow for high orientation of the fibres. The maximum total draw ratio for the fibre made from PLA2.5 was only 550%. A larger draw ratio of the fibres was obtained for fibre formed from the polymer tested last. The spinning solution of PLA12 had the most non-Newtonian rheological properties, which allowed the fibres to form in a fibre bundle with low tension (0.17 cN/tex) and the highest linear mass (158 tex). The maximum draw ratio of the PLA12 fibres was 600%.

### 3.2. Influence of the PLA Molecular Structure on the Thermal Properties of the Fibres

**Figure 3:**
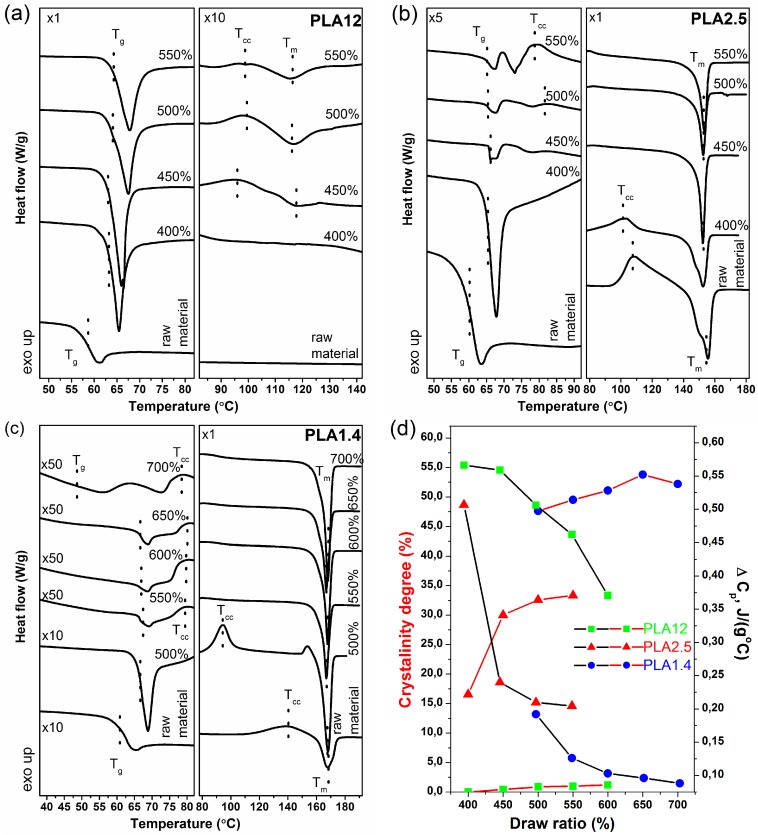
Differential scanning calorimetry (DSC) results of the studied fibres and corresponding raw materials: (**a**) PLA12; (**b**) PLA2.5; and (**c**) PLA1.4; (**d**) Relation between the calculated degree of crystallinity and the change in heat capacity (Δ*Cp*) for samples spun at different draw ratios.

The thermal characteristics of the preformed PLA fibres were analysed by the use of differential scanning calorimetry. In Figure 2, the comparison of the DSC thermographs of the studied fibres and the raw polymer is presented. Additionally, Figure 3d shows the relation between the calculated degree of crystallinity and the change in heat capacity (Δ*Cp*) for fibres drawn at different ratios. According to the literature, the degree of crystallinity was calculated using the following equation:

(2) $$\chi_{C}=\frac{\Delta H_{\mathrm{cc}}-\Delta H_{m}}{\Delta H_{100\%}}100\%$$

where Δ*H*_cc_ is the cold crystallisation enthalpy, Δ*H*_m_ is the melting enthalpy, and Δ*H*_100%_ is the melting enthalpy of 100% crystalline PLA, which is equal to 93.1 J·g^−1^ [25].

The first studied materials were fibres obtained from PLA12, which is characterised by high d-lactide isomer content. In Figure 3a, it is clearly shown that this polymer is a typical amorphous raw material without a melting point and with the only a thermal characteristic of a glass transition temperature. According to the literature, PLA containing d-lactides in the range 10%–30% is difficult to crystallise during technological processes and is mainly used for the plasticisation of polylactide materials. During the drawing process in the wet spinning method, the polymer chains of PLA12 begin to orient, and the presence of an endothermic peak slightly above the glass transition temperature is observed for all studied fibres. This peak corresponds to the relaxation of the polymer ordering during stretching in the drawing process. The ordering of the polymer chain during the wet spinning process was confirmed by the increase in glass transition temperature from 59.8 to 66.3 °C. Additionally, for the samples stretched at a draw ratio above 450%, two minor peaks on the thermogram were recorded, which shows the crystallisation of the studied material. The first peak is an exothermic peak located between 90–100 °C that corresponds to cold crystallisation, while another peak is observed at 115 °C that corresponds to the melting point of the ordered form of the polymer. The crystallisation of PLA containing 12% d-lactide isomer is observed, but the corresponding calculated degree of crystallinity is insignificant in a quantitative point of view. The maximum value of the calculated degree of crystallinity was 1.5%. 

The next studied and processed polymer was PLA2.5. This polylactide is a semicrystalline polymer that clearly shows two peaks on the recorded thermogram, where the first corresponds to cold crystallisation and the second corresponds to the melting point. The processing of the polymer during wet spinning and the orientation of obtained fibres during the drawing process influenced the increase in the glass transition temperature. Additionally, the presence of an endothermic peak around the *T*_g_, corresponding to the relaxation of the ordered polymer chains, confirmed the orientation of the fibre material during the wet spinning process. As presented in Figure 3d, the change in the degree of crystallinity and change in the heat capacity as a function of draw ratio confirm the ability of PLA2.5 to crystallise in the wet spinning process. 

For the polymer studied last, the results obtained using DSC clearly show the strong ability of PLA1.4 to crystallise in the drawing process. Glass transition and cold crystallisation points are observed for fibres formed at a draw ratio of 500%. For fibres drawn at higher draw ratios, only the melt transition was observed. The glass transition and cold crystallisation points were visible only after magnification of the appropriate thermogram. Notably, for fibres stretched at a draw ratio of 700%, a strain-induced endo–exo transition was observed in the temperature range of 40–80 °C, and the strain-induced glass transition temperature was approximately 50 °C. This phenomenon confirms the high orientation and crystallinity PLA1.4 created during the wet spinning process with high draw ratios, as presented in Figure 3d. 

### 3.3. Influence of the PLA Molecular Structure on the Crystalline Structure of the Fibres

**Figure 4:**
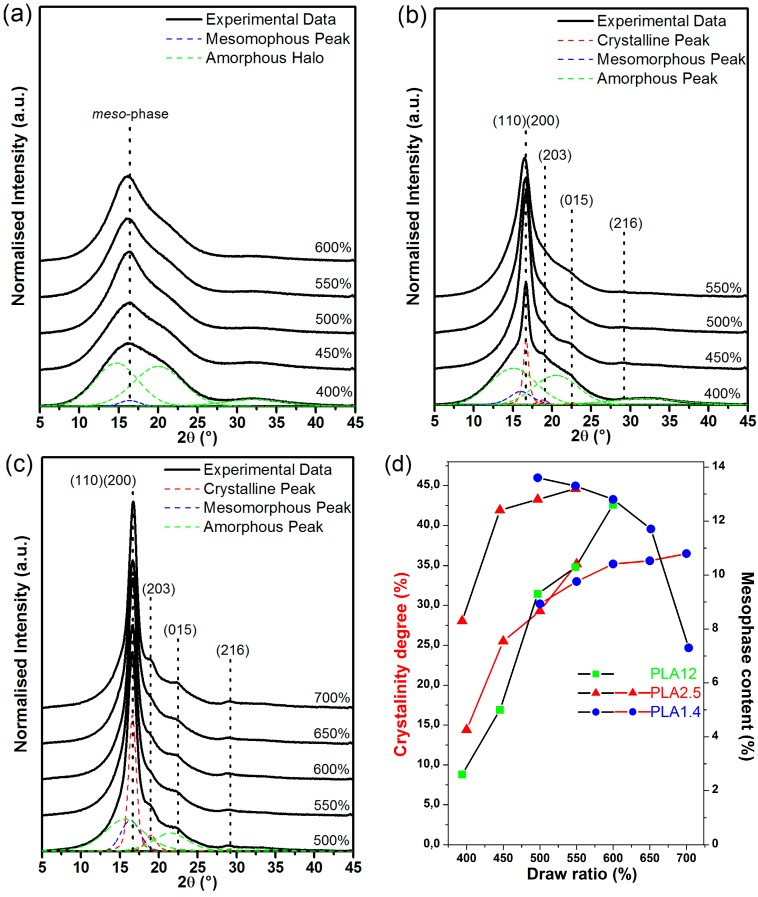
Wide-angle X-ray diffraction (WAXD) results of the studied fibres and corresponding raw materials: (**a**) PLA12; (**b**) PLA2.5; and (**c**) PLA1.4; (**d**) Relation between the calculated degree of crystallinity and the mesophase content for samples spun at different draw ratios.

The crystalline structure of fibres obtained from various types of PLA was investigated by the use of the wide-angle X-ray diffraction scattering method. The results presented in Figure 4 show the influence of the draw ratio on the crystalline structure of PLA.

The recorded X-ray diffractograms for PLA12 fibres, containing the highest content of d-lactide isomer, clearly show the tendency for this amorphous material to order (Figure 4a). All diffractograms are dominated by a single peak at approximately 16.5° superimposed on the amorphous halo, which suggests that the peaks originate predominantly from the mesomorphic form (intermediate form of ordering polymer chains between amorphous and crystalline form) rather than the well-developed crystalline phase. This conclusion is supported by DSC thermograms in which a marked post-*T*g endothermic effect followed by an exothermic peak and slightly visible peaks, corresponding to cold crystallisation and the melting point, were observed. 

In the recorded diffractograms of the PLA2.5 fibres formed at various draw ratios, excluding the diffraction peak originating from the mesophase, the diffraction peak located at 2θ = 16.5° corresponds to the (110) and (200) lattice plane of the α and α’ crystalline form of PLA, respectively (Figure 4b). Additionally, low-intensity peaks located at 2θ = 18.8°, 22.3°, and 28.8° were assigned to the reflection from the (203), (015), and (216) crystallographic planes, respectively, and intensified with increasing draw ratio. The presented WAXD results of the semicrystalline structure of the fibres formed from PLA2.5 confirms the results of DSC.

The final recorded X-ray diffractograms were obtained for fibres formed from PLA1.4 (Figure 4c). All presented WAXD curves showed clear diffraction peaks located at 2θ = 16.5°, 18.8°, 22.3°, and 28.8° corresponding to the (110)/(200), (203), (015), and (216) lattice planes of the α or α’ form of PLA. The intensity of the peaks may suggest the significant order of the supramolecular structure of the studied material. Comparing the obtained PLA1.4 results with the results obtained for other samples, it can be concluded that the fibres of this polymer were the most crystalline. 

A quantitative structural analysis of the investigated samples was obtained by the deconvolution of the patterns of the amorphous halo and the crystalline and mesomorphic peaks. For this analysis, the experimental data were fitted by a composite of the Gauss and Lorentz functions calculated using the WAXSFIT software based on Hindeleh and Johnson’s method [26]. The shapes of the amorphous halo and the mesomorphic and crystalline peaks were selected according to the model proposed by Stoclet et al. [27].

The crystalline phase and mesophase content in the investigated fibres were calculated using the following equation:

(3) $$\chi_{C}=\frac{A_{C}}{A_{C}+A_{A}}100\%$$

where *A*_A_ and *A*_C_ are the integral intensities of the amorphous halo and the peaks originating from the crystalline or mesomorphic phase, respectively. The degree of crystallinity and mesophase content as a function of the draw ratio of the studied fibres are presented in Figure 4d.

The mesomorphic phase of PLA is detectable in the structure of all studied fibres. PLA12 is the only form with polymer chain ordering. For this reason, only one X-ray diffraction peak at 16.5° is clearly visible in the diffractograms of these materials (Figure 4a). The mesophase content increases with increasing of draw ratio, which was expected. The calculated maximum value of the mesomorphic phase content was approximately 13% for PLA12. 

The increasing mesophase content as a function of draw ratio was also observed for PLA2.5. This parameter value characterises the mesomorphic order of polymer and varies from 2% at a draw ratio of 400% up to 13.2% at a draw ratio of 600%. The supramolecular structure of the fibres formed from PLA2.5 is also crystalline, which also shows an increase in the mesophase content with increased draw ratio. 

For the final investigated fibres formed from PLA containing the lowest content of d-lactide isomer, a decrease in the mesophase content with increasing draw ratio was observed. For the fibres formed from PLA1.4, the disappearance of the mesomorphic structure is accompanied by a significant increase in the degree of crystallinity. The obtained results suggest that the content of d-lactide isomer and molecular weight affect the creation of the mesophase. For the polymer that contains the lowest content of d-lactide, the mesophase is more pronounced in the fibres formed at lower draw ratio. At the higher degree of stretching that took place at higher draw ratio, the ordering of the mesophase into the crystalline form was observed. Increasing the content of d-lactide isomer and the molecular weight of the raw material results in a simultaneous increase in the crystalline phase and the mesophase. There are two ordered forms of the polymer, and the maximum crystallinity is lower than that of materials with lower d-lactide content and lower molecular weight.

Another characteristic feature of the structure formed under different draw ratio conditions was detected by an analysis of the lattice length (d-spacing) calculated for the crystalline material according to Bragg’s equation:

(4) $$d=\frac{\lambda }{2\sin\theta }$$

where λ is the wavelength of the X-ray source (0.15418 nm) and θ is the angle of reflection (half of the 2θ peak position). 

The lattice length was calculated for the most intense diffraction peaks corresponding to the (110)/(200) planes. For the PLA characterised by a molecular weight *M*_w_ of approximately 112 kg/mol, *M*_w_/*M*_n_ of 1.46, and content of d-lactide equal to 2.5%, the calculated lattice length decreased slightly with an increase in the draw ratio (DR) from 0.534 nm at DR 400% to 0.531 nm at DR 550%. The disorder-to-order phase transition of PLA was insignificant for this polymer. It should be assumed that the crystalline structure of these polymer materials is the α’ form. 

More evident changes in the crystalline structure were observed for PLA characterised by a *M*_w_ of approximately 59 kg/mol, *M*_w_/*M*_n_ of 1.29, and content of d-lactide equal to 1.4%. The calculated lattice length for PLA1.4 fibres decreased less that of PLA2.5 as a function of draw ratio from 0.535 nm at DR 700% to 0.529 nm at DR 700%. In this case, the disorder-to-order phase transition of PLA was observed. At low draw ratios, the crystalline structure was the α’ form, and above a draw ratio of 650%, it was the ordered α form. This is a critical observation in the mechanical properties of the fibres. In the works referred to earlier, the ordering of the crystalline structure of the polymer had a negative impact on the strength of the material [15,23].

### 3.4. Influence of the PLA Molecular and Supramolecular Structure on the Mechanical Properties of the Fibres

The mechanical properties of the studied PLA fibres were characterised by two typical parameters, stress at break and strain at break. The changes in the mechanical properties of the studied fibres formed at different draw ratios are presented in Table 4.

In Table 4, the mechanical parameters of the fibres (such as stress at break) as a function of draw ratio are presented. As is clearly shown, the increase of draw ratio influences the properties of the studied materials—for example, increase of stress at break and decrease of linear mass. Physical parameters such as stress and strain strongly depend on the supramolecular structure created by stretching the fibre at various draw ratios. The increase of draw ratio effect on orientation of polymer chains in fibre structures, which is connected with their mechanical properties. As it turns out, the molecular structure of the raw polymer is also important [28,29]. For PLA12, where the drawing process creates the mesophase, the stress-at-break value increased with increasing draw ratio from 5.41 cN/tex at DR 400% to 18.63 cN/tex at DR 600%. It is, therefore, apparent that the increase in the strength of the fibres depends on the increase in the content of the mesophase of the PLA material, as presented. The strain-at-break value also increased with increasing draw ratio, but only up to 500%. Above DR 550%, the fibres were less capable of elongation. Most likely, mesophase contents higher than 10% cause an increase in stiffness and impart fragility to the fibre.

Additionally, for PLA with reduced d-lactide isomer content, the mechanical properties are improved by stretching the fibres to the same draw ratio during manufacturing. The values of strain at break and stress at break significantly decrease for draw ratios above 500% for PLA2.5 and above 600% for PLA1.4. This is due to both the increase in crystallinity and the more orderly arrangement of the chains, implying a marked decrease in lattice length. For PLA1.4, the change in the lattice length value suggests that the disorder-to-order crystalline phase transition is accompanied by a decrease in the mechanical properties. 

## 4. Summary

The performed experiments and detailed analyses clearly show the dependence of the molecular ordering of PLA during the wet spinning process on the molar mass and d-lactide content. The investigation also allows for the analysis of the influence of the PLA molecular structure on the technological conditions of the wet spinning process and physical properties of the final material. The most important conclusions are as follows:

1. For PLA, not only the molar mass but also the d-lactide content affects the rheological properties of the spinning solution. The viscosity of a spinning solution is increased with increasing molar mass, but this effect may be changed by increasing d-lactide isomer content. Moreover, the spinning solution prepared from the polymer that contained the highest d-lactide content (12%) was the most non-Newtonian solution, but the rheological properties of the spinning solution prepared from PLA that contained only 1.5% of d-lactide were close to those of a Newtonian fluid.
2. The molecular structure of PLA influences the spinning solution and spinning parameters, such as draw ratio. The fibres could be manufactured at the highest draw ratio using the PLA with the lowest d-lactide content and lowest molar mass.
3. The draw ratio affects the molecular ordering of PLA fibres. For the amorphous polymer containing 12% d-lactide isomer, only the mesophase was created, but for the polymers similar to the homopolymer, a mesophase, a crystalline phase, and a disorder-to-order phase transition as a function of draw ratio were observed.
4. The creation of the ordered α form at high draw ratio decreased the mechanical parameters of the fibres, which was expected.

## Figures and Tables

**Table 1:** The characteristic of raw materials.

Sample	Nature works symbol of PLA	*M*_w_ (g/mol)	*M*_w_*/M*_n_	Contents of d-lactide (%)
PLA12	4060D	119,000	1.40	12
PLA2.5	2002D	112,600	1.46	2.5
PLA1.4	6201D	59,100	1.29	1.4

**Table 2:** Rheological parameters of the studied polylactide solutions of different concentrations.

PLA	Polymer concentration (%)	Rheological parameters	
		*k* (Pa·s)	*n*
PLA12	25	22.76	0.753
PLA12	26	30.88	0.749
PLA12	27	58.18	0.719
PLA2.5	23	33.74	0.891
PLA2.5	24	56.98	0.877
PLA2.5	25	71.41	0.862
PLA1.4	28	20.49	0.962
PLA1.4	29	32.22	0.947
PLA1.4	30	52.88	0.878

**Table 3:** Fibre formation conditions from selected PLA solutions.

Fibre	Total draw ratio (%)	Linear mass (tex)	Tension of fibre bundle (cN/tex)
PLA12-F1	400	158.00 (2.09 *)	0.17
PLA12-F2	450	98.33 (1.85)	0.39
PLA12-F3	500	92.83 (1.25)	2.37
PLA12-F4	550	92.00 (1.01)	5.51
PLA12-F5	600	80.33 (0.90)	12.45
PLA2.5-F1	400	121.00 (1.43)	1.22
PLA2.5-F2	450	112.67 (1.09)	2.26
PLA2.5-F3	500	90.67 (1.07)	4.54
PLA2.5-F4	550	72.67 (0.79)	10.25
PLA1.4-F1	500	96.00 (1.04)	0.35
PLA1.4-F2	550	91.33 (0.93)	6.13
PLA1.4-F3	600	85.33 (0.69)	8.67
PLA1.4-F4	650	68.33 (0.52)	12.57
PLA1.4-F5	700	60.33 (0.46)	15.25

* The coefficient of variation is in brackets.

**Table 4:** Mechanical properties of fibres formed from the selected PLA at various conditions.

Fibre	Total draw ratio (%)	Linear mass (tex)	Stress at break (cN/tex)	Strain at break (%)
PLA12-F1	400	158.00 (2.09 *)	5.41 (6.75)	2.61 (11.66)
PLA12-F2	450	98.33 (1.85)	6.93 (5.88)	4.12 (15.69)
PLA12-F3	500	92.83 (1.25)	15.33 (2.28)	28.36 (2.68)
PLA12-F4	550	92.00 (1.01)	17.05 (4.18)	28.25 (2.70)
PLA12-F5	600	80.33 (0.90)	18.63 (4.39)	26.34 (2.78)
PLA2.5-F1	400	121.00 (1.43)	4.32 (7.46)	1.92 (8.19)
PLA2.5-F2	450	112.67 (1.09)	21.01 (4.73)	32.42 (5.97)
PLA2.5-F3	500	90.67 (1.07)	22.22 (5.23)	23.11 (4.24)
PLA2.5-F4	550	72.67 (0.79)	20.11 (4.70)	19.65 (4.32)
PLA1.4-F1	500	96.00 (1.04)	18.21 (4.07)	25.97 (4.50)
PLA1.4-F2	550	91.33 (0.93)	21.39 (5.64)	22.38 (3.31)
PLA1.4-F3	600	85.33 (0.69)	26.05 (4.50)	20.97 (3.84)
PLA1.4-F4	650	68.33 (0.52)	24.84 (4.14)	20.15 (4.08)
PLA1.4-F5	700	60.33 (0.46)	23.16 (9.43)	18.21 (4.07)

* The coefficient of variation is in brackets.
